# Editors’ Choice—Perspective—Deciphering the Glycan Kryptos by Solid-State Nanopore Single-Molecule Sensing: A Call for Integrated Advancements Across Glyco- and Nanopore Science

**DOI:** 10.1149/2754-2726/ad49b0

**Published:** 2024-05-24

**Authors:** Megan E. Kizer, Jason R. Dwyer

**Affiliations:** 1 Department of Chemistry, Brown University, Providence, Rhode Island 02912, United States of America; 2 Department of Chemistry, University of Rhode Island, Kingston, Rhode Island, 02881, United States of America

**Keywords:** interface modification, nanoscale materials, sensors, surface functionalization, surface modification, glycomics, nanopore

## Abstract

Glycans, or complex carbohydrates, are information-rich biopolymers critical to many biological processes and with considerable importance in pharmaceutical therapeutics. Our understanding, though, is limited compared to other biomolecules such as DNA and proteins. The greater complexity of glycan structure and the limitations of conventional chemical analysis methods hinder glycan studies. Auspiciously, nanopore single-molecule sensors—commercially available for DNA sequencing—hold great promise as a tool for enabling and advancing glycan analysis. We focus on two key areas to advance nanopore glycan characterization: molecular surface coatings to enhance nanopore performance including by molecular recognition, and high-quality glycan chemical standards for training.

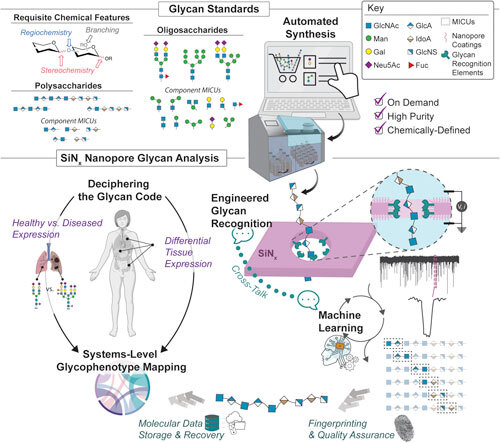

## Highlights

- Glycans are vital, abundant biomolecules needing better chemical analysis tools.
- Overview and prospects of nanopore sensing for glycan analysis.
- Improved glycan synthesis and machine learning are needed for nanopore glycomics.
- Better surface coatings and molecular recognition will help nanopore glycomics.
- Discussion of automated glycan synthesis for glycoscience and nanopore sensing.

Glycans are the most abundant natural products and play a host of vital biological roles including in communication and protection across organisms, energy storage, structural scaffolding, and post-translational protein modification.^1–5^ Glycans are also information carriers, food additives, and clinical therapeutics.^1–6^ Yet our current understanding of glycan function is limited compared to other biomolecules such as DNA and proteins.^7^

Glycobiology, itself, imposes a profound information gap because glycans are not directly genetically encoded. Rather, glycan biosynthesis is driven by glycosyltransferase (GT) activity controlled by biochemical and environmental factors. Thus, DNA sequence does not provide a reference key for glycan sequencing as it does for directly genetically encoded proteins. Instead, glycan sequence tantalizes as the doorway to exploring both glycan functional roles and detailed origins.

Even if the nanopore devices and methods currently useful for DNA sequencing could be simply applied to glycan profiling—and they cannot—the sheer number of possible monomers (120 for eukaryotes and >800 for prokaryotes) defies easy analysis.^8^ The diversity of their complex polymeric forms compounds this challenge. Sequencing free glycans includes specifying the stereochemistry at each ring position; glycosidic linkage pattern; monomer form (pyranose or furanose) and order; and branching pattern (*cf* unbranched DNA and protein chains). Preparation of glycans by synthesis or purification requires control over all of these features and thus also defies tractability. The very chemical structure of carbohydrates that enables such distinct information-carrying channels also hinders progress towards decoding carbohydrate-based communication. Much of the carbohydrate biomass results from glycoconjugates, a more complicated sequencing context: to analyze a glycoprotein, protein and glycan sequences and glycan location must all be determined.^9^

Total sequencing—all monomer identities, forms, locations (encompassing order, branching), and connectivities—is the ultimate technical aim of glycan sequencing. The goal of “decoding” carbohydrate-based communication and information transfer, however, especially in the biological context, admits parsing sequence into “minimal information-containing units” (MICU), which can be thought of in analogy to epitopes, albeit initially in a nanopore sensing context rather than a biological function context. MICU identification (or sequencing) is a reduced burden, just as genotyping presents lower barriers than DNA sequencing.^10^ It definitively eases chemical synthesis and conventional characterization burdens when generating glycan standards. A MICU-level focus provides a foothold for analysis by molecular recognition. Moreover, the functional and organizational importance of MICUs in relation to epitopes might be leveraged to extract a richer view of the glycome than might be imagined from a less biologically integrated total sequencing approach.

The absence of an amplification method for glycans, in contrast to PCR for DNA, means that sample amounts and limits of detection must be compatible. Conventional instrumental approaches for analysing glycans include capillary electrophoresis,^11–13^ glycan microarrays,^14–18^ nuclear magnetic resonance (NMR)^19^ and mass spectrometry (MS)^20–22^ (including hyphenated MS approaches).^23–25^ MS cannot always differentiate between glycans with identical molecular masses and glycans are refractory to analysis by MS, so expert users are required.

Epitope detection, molecular recognition, and interaction-based analysis have been implemented using biochemical and chemical approaches. Biochemical methods have heavily relied on existing carbohydrate-binding molecules, typically lectins, antibodies, and carbohydrate binding modules (CBMs). Carbohydrate-binding aptamers have had limited success and limited transfer to a commercial or clinical setting.^26–29^ Overall, the challenges that arise with engineering biochemical methods towards glycan recognition are the inherently low affinity and low selectivity of currently used biomolecules to carbohydrates. Chemical methods rely on the use of interactions of boronolectins and boronic acids,^30,31^ biomimetic macromolecules,^32^ and instrumental approaches such as capillary electrophoresis. Fluorescent labelling for optical detection of the naturally optically inactive carbohydrates adds to the analysis workflow and complexity.

These difficulties provide vital context for the challenges that must be addressed in glycan analysis—either by ongoing advancements using conventional approaches, or by new approaches. The considerable successes of conventional glycan analysis must be acknowledged, especially since they will be crucial for supporting the development of new ones.^12,13,16–18,22^ Indeed, the foundational and practical barriers to glycan preparation, characterization, availability, and use cannot be avoided with a focus only on nanopore technology improvements. Thus, while the focus of this Perspective is how to develop nanopore technology as a compelling addition to the glycan analysis toolbox, that focus must be tightly integrated with what concomitant, supporting glycoscience advances are needed. Our emphasis is therefore on what improvements are needed in both nanopore science and glycoscience in order to deliver grand and aggressive analysis aims, rather than on a forensic examination of current nanopore capabilities on their own, or in comparison to conventional approaches.

**Figure 1.**
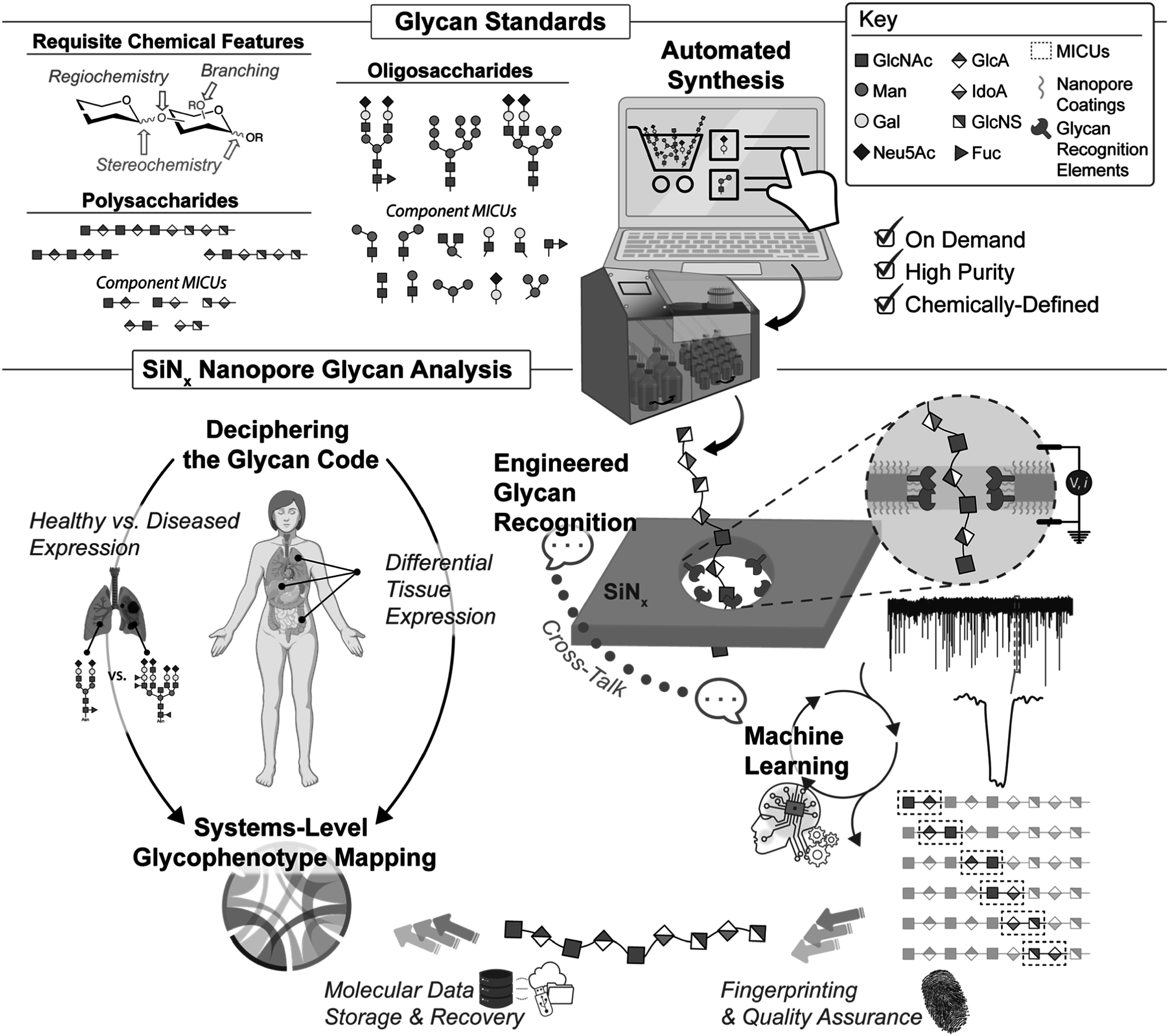
Glycans are key biological molecules providing a host of functions in nature that can be leveraged for a myriad of health and technological applications. Cracking the glycan code remains a formidable challenge, though, because glycan chemical and structural complexity poses significant technical difficulties for chemical analysis. Solid-state (SiN_x_) nanopore sensing is a promising candidate for next-generation glycan analysis. Nanopores offer direct single-molecule manipulation and sensing capabilities, and engineered surface coatings can improve performance through various mechanisms including glycan recognition. A concerted effort across disciplines is required to advance the technique: by improving the availability of high-quality, on-demand glycan standards (from minimal information containing units (MICUs) up to polymers); by design of higher performance coatings including molecular engineering of improved glycan recognition agents; and by using machine learning in concert with glycan standards and surface coatings to inform interative experimental improvements that target enabling technological applications of glycans and driving better understanding of these key biomolecules across biological systems.

Given the challenges posed by glycans, it is unsurprising that the high-priority recommendations of the 2012 National Research Council report *Transforming Glycoscience: A Roadmap for the Future* ^33^—“the development of transformative methods for the facile synthesis of carbohydrates and glycoconjugates…, [and] of transformative tools for detection, imaging, separation, and high-resolution structure determination of carbohydrate structures and complex mixtures”—remain pressing. While nanopore glycoscience will depend heavily upon hard-won advances in conventional instrumentation, analysis, and glycan synthesis, the transformative potential of nanopores is through their fundamentally different operation.^34–38^ Nanopores expand the glycoscience toolkit with single-molecule sensitivity^39^ without sample labelling, with conceptual simplicity of design and operation, and with practicable compatibility with existing manufacturing methods. They inspire visions of a low-cost, low-barrier tool for glycomics that could support interdisciplinary research and applications (Fig. 1).

Nanopores are nanoscale channels formed through insulating membranes.^34–38,40–46^ There are a number of different nanopore sensing platforms—protein, polymer, pipette, molecular, and thin-film (down to 2D materials) solid-state—that have history and promise for glycan analysis. Each type offers different opportunities and challenges in terms of geometry and material composition—including tunability of both—and of fabrication and deployment configuration.^34–38,40–46^ The elements of nanopore sensing performance that are determined by nanopore surface chemistry^6,42,47–50^ can be partly or completely decoupled from their underlying nanopore material composition when suitable surface decoration methods are available. Different nanopore types will generally require different coating or decoration methods and we refer the reader to the literature, including comprehensive reviews.^35,38,40,42,51–55^ In this focused Perspective article, we feature size-tunable (∼1–100+ nm-diameter, ∼10 nm-long) solid-state nanopores in thin-film silicon nitride (SiN_x_) membranes because SiN_x_ offers conventional microfabrication compatibility with implications for the potential manufacture, at scale, of sophisticated multipore devices with integrated electronics.

Passage of a single molecule near or through a suitable electrolyte-filled nanopore perturbs the distribution and voltage-driven transit of electrolyte ions and can produce a detectable change in ionic current.^34,56–58^ Beyond revealing the presence of a single molecule, the ability to resolve the passage of a polymer down to unique monomer-level-current perturbations would be salubrious for sequencing efforts. Such resistive-pulse (or “event”) sensing is the most prevalent implementation of nanopore sensing. Events (or event substructures) are typically described by their mean magnitude and duration, with machine learning approaches^59^ often invoking additional signal parameters. Analysis of current fluctuations and current switching between states can be especially fruitful when nanopores have been enhanced with molecular recognition agents that cause (transient) analyte binding or other interactions.^34,41^ In the limit of long-lived binding or of an ensemble of surface-bound analytes, signals transition from discrete resistive pulses to quasi-steady-state changes in nanopore conductance (or rectification for asymmetric pores).

## Current Status

### Nanopore glycan analysis

Nanopore glycan analysis has had a far more measured pace of development than was seen for nanopore DNA sequencing,^35,58^ as has recently been critically reviewed.^60^ A small collection of experiments over more than two decades has used different nanopore types with and without surface-bound carbohydrate capture agents (e.g. lectins and boronic acids) and chemical labelling of analytes to characterize a range of glycan samples and characteristics.^35,61–83^ Recent glycoscience work with SiN_x_ nanopores has resolved hyaluronic acid biopolymer molecular weights,^67,68,80^ differentiated between multiple survey sets of samples by chemical composition,^77^ and demonstrated the promise of combining solid-state nanopore sensing with machine learning.^75,76^ The indispensability of high-quality glycan standards for machine learning training in nanopore glycoscience was underscored in work that differentiated between glycans by unique monomer composition.^76^ A bottom-up approach for training machine learning used phenylboronic acid labelled protein nanopores to transiently interact with the readily available monomer and disaccharide glycans standards and *cis*-diol fruit extracts.^69,78,83^ Extending this approach to monomers digested from a glycopolymer—or perhaps within an intact glycopolymer—would require appreciable further development, but the approach is nevertheless compelling and contextualizes enabling concepts and approaches.^84^

Analytes transiting small nanopores are subject to nanoconfinement and interactions with the nanopore surface. Electrolyte solution structure will also be perturbed by the surface and nanoconfinement. Surface coatings and treatments allow chemical tuning of these effects, can add functionality such as resistance to clogging, and can make “smart(er) nanopores” with integrated molecular recognition agents. Coatings can also finely tune nanopore dimensions. Several coating chemistries have been reported for the SiN_x_ nanopores that this Perspective focuses on,^38,51–54,85^ but considerable potential in using surface-coated nanopores remains unexplored and untapped. We find that photohydrosilylation chemistry offers a simple, reliable, and robust route to covalent attachment of an organic monolayer of a range of commodity chemicals to SiN_x_ nanopores that subsequently supports coupling other species, including proteins, to the coating.^35,85^ Having such accessible and straightforward methods reduces barriers to more rapidly exploring the effects of surface film composition, thereby establishing principles that might be implemented in optimized devices using other surface decoration approaches or other nanopore membrane materials.

### Glycan standards and recognition

Analytical standards are needed to develop nanopore glycan analysis, just as readily available, high-purity, well-characterized DNA standards underpinned the development of nanopore single-molecule DNA sequencing. Glycan standards face stringent requirements: high-purity, homogeneity, chemical definition (i.e. with the correct monosaccharide composition and sequence, stereochemistry of monomers and linkages, *etc*). To be practical, they must be readily accessible in sufficient amounts at low cost (as with synthetic DNA). Obtaining glycans as standards directly from biological sources is not currently viable. Samples are heterogeneous, may exist biologically in unmanageably small quantities, and certain glycans from particular conjugate classes (e.g. mucins) cannot be accessed due to our current inability to cleave intact glycans from the biomolecular support. Careful consideration must also be given to the reducing sugar remaining in equilibrium between its closed ring and open chain forms. Beyond the sensing context, nanopore methods may yield gains for glycan separations in the face of such challenges. However, at present, the laborious, complex, low-yielding, and costly set of conventional steps for chemically synthesizing glycan standards limits the availability—and thus the practicable diversity—of glycan standards.

Carbohydrate recognition, and particularly our ability to engineer better recognition elements, has overall remained quite stagnant through the past 20 years. The most widely utilized molecular recognition agents for the detection of carbohydrate epitopes are glycan binding proteins (GBPs), particularly lectins and antibodies. Lectins are a broad class of proteins, often multimeric, that selectively recognize carbohydrate epitopes. While other recognition elements have been developed, including boronic acids (boronolectins), molecularly imprinted polymers (MIPs), and aptamers, there remain individual limitations that prevent them from being substantively adopted. There have been numerous engineering efforts to improve GBP glycan recognition. Many of these attempts have failed to yield significant gains in protein specificity or binding affinity to the desired target,^86^ and the glycobiology community still relies on the generally available wild-type lectin and antibody classes of GBPs.

From efforts to avoid the limited molecular engineering tractability of existing lectin and antibody GBP scaffolds, two new classes of novel GBPs have recently been developed as promising alternative GBP scaffolds. “Lambodies”^87,88^ are carbohydrate-binding antibodies generated by immunizing lamprey fish embryo with low molecular weight mammalian saccharide antigens (mono- to tetrasaccharides). They show excellent selectivity for the glycan antigen target. Multimeric organization—lambody dimers coupled to an IgG stalk domain—offers higher avidity, albeit with larger size. Directed evolution of a DNA-binding protein scaffold was used to generate a different new class of disaccharide-binding GBPs^89^ that are small (7 kD), highly thermostable, and easy to produce in high yield by recombinant methods, albeit with some off-target recognition challenges.

## Future Needs and Prospects

The ultimate aim of developing nanopore technology for glycomics should extend beyond fully characterizing the analytical target. It should include commercialization and end-use considerations that often see the invocation of the terms streamlined, automated, plug-and-play, hand-held, and error-tolerant, among others.^58^ Integration of the (nanofluidic) nanopore into a microfluidic device emerges as a natural part of the technological development.^90–94^ Microfluidic capabilities could be used to process samples before sensing when desired—by separating complex mixtures (e.g. by dielectrophoresis), enzymatic digestion, preconcentration (e.g. again by dielectrophoresis), and chemical labelling and derivatization of analytes including chemical transformation (e.g. permethylation) and use of molecular and polymeric binding tags to give nanopore signal contrast or enhancement by sterics or physicochemical property changes.^79,95–100^ They could be used to install on-the-fly custom nanopore coatings or, more ambitiously, to synthesize glycan standards on-demand for real-time device calibration. Hyphenated instrumental approaches such as nanopore-MS captivate the imagination:^101^ complementary information could yield to better understanding glycan structure and function.

Leveraging the molecular-level control and achievable chemical and structural diversity of nanopore surface compositions is vital to improving nanopore glycomics.^6,35,40,58,82,102^ One approach is a change of nanopore membrane material to existing or yet-to-emerge candidates, and surface chemical tuning—independent of underlying nanopore composition—will aid generality. Different nanopore choices may impose material and geometric constraints on how to apply surface coatings and which types will have their function preserved, for example from anisotropy in chemical reactivity and structure and vanishing thicknesses of 2D materials. Coatings have been largely underexploited in the nanopore sensing domain and have the potential to serve a profound and active role in uncovering mechanisms to exploit in improving performance. There are a variety of different choices resulting in films with different chemical and physical structures and properties; stabilities; mechanisms and degree of attachment to the pore walls; and degrees of coverage.^35,38,40,42,51–55^ Such factors can impinge upon nanopore sensing performance in complex ways so that ongoing surface-chemistry-centric studies will be important. Nanoconfinement, in addition, has the potential to amplify the effect of what might otherwise be innocuous coating choices.^50^ Nanopore surface coating selection and design should be closely integrated with nanopore glycan analysis.^58^ For ease of presentation, we artificially partition these connected pursuits but emphasize that throughout, the questions “how might nanopore glycan sensing better inform coating choice and design,” and “how might nanopore coatings improve nanopore glycan sensing and characterization,” should be foregrounded.

### Nanopore glycan analysis

The continued development of nanopore glycan characterization is central but should synergistically help benchmark and guide the ongoing development of glycan synthesis, glycan molecular recognition, and surface chemical approaches. Nanopore DNA sequencing was developed using existing on-demand, high-quality synthetic DNA oligomers of desired sequences (aided by the limited natural DNA base diversity and linkage uniformity): glycan standards synthesis and availability will need to be developed *alongside* the nanopore measurements. We foresee an iterative process, beginning with surveys across different available sets of glycans—varying by size and composition (MICU-level framing through polymer), and with the high purity necessary to extract relationships between signals and molecules. While surface coatings that support molecular recognition in small synthetic MICUs will drive longer-term molecular-recognition of MICUs (or even epitopes) in larger biopolymers, and should be a major research focus, experiments focusing on translocating glycans through nanopores without engineered surface interactions should also be performed. Such “free translocation” studies will be important for developing surface coatings that enhance operational ease and reliability, suppress fouling and increase useful sensing lifetime, and as controls for molecular recognition studies. Ongoing efforts by others in improving uncoated nanopore fabrication will be important, as will employing surface coatings to tune nanopore size.^85,102–107^ The use of nanopore arrays may yield benefits both by producing higher throughput and allowing an exploration of tolerance to—or benefits of—fabrication nonuniformity. The use of photohydrosilylation for surface functionalization allows for different surface coatings to be installed in each uniquely optically addressable SiN_x_ pore in nanopore arrays. A library of nanopore signals from various glycan standards will allow for qualitative consideration and comprehensive supervised machine-learning analyses. Adopting rigorous machine learning best-practices will be essential for extracting meaningful information.^59^ At this stage of the iterative process, new glycan standards should be selected: to try to maximize signal differences if existing ones are slight; to test for the minimal detectable contrast differences (e.g. composition or linkage); or to evaluate new solution conditions, nanopore sizes, or GBPs. With a larger database of results from hypothesis-, rational- and data-driven choices of glycans, sensing conditions, and sensing methodologies, it is hoped that theory and simulation of nanopore glycan sensing will play an increasing role in this iterative development process.

Coatings of up to a monolayer of small molecules (through to polymers) already serve a number of important goals, including: diminishing the impact of choice of nanopore membrane material on the nanopore surface (subject to the availability of suitable coating methods); depending on coverage and properties, shielding the underlying nanopore surface from solution and analyte; depending on functional group, allowing electrostatic and electroosmotic tuning; and providing an anchor point for film extension or coupling of additional molecules such as proteins.^35^ Lipid-bilayer-coated solid-state nanopores offer biomimicry with a mobile coating that can be decorated with other biomolecules to control interactions with the analyte.^52,108^ Coupling a suitable protein (including a protein nanopore) or molecular machine offering active or passive high-resolution control over glycan nanopore passage—as for nanopore DNA sequencing—directly to a solid-state nanopore^109,110^ would be a compelling goal of both protein design and surface functionalization.

The next steps for nanopore surface coatings in glycomics will include:

(1) Installing and characterizing functional molecular recognition agents for nanopore sensing, both existing agents and, iteratively, those developed for nanopore glycomics.
(2) Systematically exploring homogeneous surface coating preparations, properties, and performance to improve the end-user experience. For example, are there film design principles to replicate >1 million single biopolymer events as seen in uncoated, but chemically-tuned, SiN_x_ nanopores?^102,103^ How do film hydrophobicity, sterics,^6^ fluidity and reversibility affect glycan sensing performance and the stability of the coating and underlying SiN_x_? Evaluating stability requires the unglamourous, but essential work of lifetime testing.
(3) Developing methods to form and characterize mixed-composition surface coatings tailored for multiple functions, *e.g.* surface passivation against etching of underlying SiN_x_ or against biofouling while also supporting molecular recognition. Different coatings inside the nanopore and on the planar membrane may also be useful.^42^ Results from (2) will inform this effort and may together provide better insight into the performance and tunability horizons of surface coatings under nanoconfinement.
(4) Developing methods to uniquely chemically coat each nanopore in nanopore arrays to provide greater flexibility to parallel nanopore measurements. Photohydrosilylation is poised to do this for SiN_x_ nanopore arrays.

### Glycan standards and recognition

The ultimate aim for a glycan standard is a high-purity, homogeneous sample of on-demand^111,112^ length, sequence and total structure, available at scale—drawing upon the entire glycan monomer constituent space and spanning from MICU through oligomer to polymer scale. In the next several years, the feasibility of all of those targets remains a significant challenge and a focus on MICU-level glycan standards offers several interrelated benefits, although that focus should not forestall the pursuit of oligomer and polymer targets. Thus, glycan standards should contain *at least* a MICU appropriate to addressing the particular glycan analysis challenge. For example, consider a disaccharide with the reducing end monosaccharide functionalized at the anomeric carbon such that it is “locked” into its conformation, *e.g.* Neu5Ac*α* 2–6 Gal*β*1-R. Full determination of this MICU requires monomer identities (Neu5Ac and Gal) and positions, how and where Gal is linked to Neu5Ac ($\alpha $2–6), and how Gal may be $\beta 1$-linked to the rest of the carbohydrate or to another biomolecule (R). This level of information, beyond specification of monomer identity and order, is necessary to link back to the broader carbohydrate context. Larger standards up to polysaccharides are important for validating the MICU fragment-based analysis of an unknown carbohydrate as well as for remaining a primary target length-scale for analysis. For investigating branching in intact glycans, however, the initial MICU would have to be a tri- or tetrasaccharide. Small MICU sizes aid in lowering synthetic and conventional characterization barriers, which allows for more rapid and efficient iteration between synthesis and nanopore measurements.

While the use of enzymes in standard synthesis has aided purely chemical methods in scalability and specificity of the reaction,^113,114^ glycosyltransferase enzymes are still subject to promiscuous/non-specific substrate addition and reverse transformations, and we do not have available a sufficient number of enzymes to catalyze the vast landscape of transformations needed. As such, there is great interest in the biochemical space to engineer glycosyltransferase enzymes for novel and improved glycosidic bond formation.

Small size is also suitable for exploring molecular-recognition. More broadly, the small initial MICU size also provides a convenient reference point for expanding the glycan standard size if the minimal information *providing* unit, in the nanopore readout context, needs to be larger. Thus, these small MICU studies offer tractability and efficiency in pursuit of insights from, and synergy between, the underlying aspects of the overarching goal of developing nanopore glycan analysis.

Constraining the MICU size will not be sufficient for early-stage tractability alone, however. Given carbohydrate complexity, it is reasonable to initially examine only a subset of glycan standards. They must be selected carefully to maximize the information available for constructing an underlying information base for nanopore-based glycan sensing and sequencing. The key question of *What MICUs should be made first?* will require the tight integration of exploratory survey experiments using readily available or specifically synthesized test glycan MICUs, data science and machine learning analysis, and simulations. The initial seed experiments may involve nanopore characterization of “go-to” samples well-known to practitioners in the field that may not otherwise have been chosen as part of the standard set. However, it is vital to remain anchored by the questions underlying which samples would be first worth analyzing, including: which are hypothesized to be high vs low contrast; which are hoped to either provide immediately productive glycoinformatics data from nanopore sensing or serve as an impetus for further nanopore technical development; which MICUs are hallmarks of a larger glycan structure; and whether there are samples of greater clinical urgency or impact in the moment than others.

Many of the issues of glycan recognition stem from generally low affinity and selectivity. The energetic gain of these polyhydroxylated biomolecules binding to their partner proteins is so low that it provides only modest monomeric binding affinities. This is worsened by the chemical similarity of individual monosaccharides that allows for off-target binding. In addition to these fundamental challenges, there is a dearth of information related to glycan-protein interactions. Such recognition elements generally rely on hydrophilic, ionic, and CH-*π* interactions, but the question of how to use such limited information to engineer greater affinity and specificity is still elusive. This drastically limits efforts to effectively engineer novel and better recognition elements to improve nanopore-based sequencing. Thus, it is imperative to continue to characterize and catalog the variety of glycan recognition methods and to couple such characterizations with machine learning pipelines to provide clear cut “rules” for recognition of a particular epitope or MICU. Here, nanopores offer two unique capabilities for providing insight and control over glycan molecular recognition. First, nanopore force spectroscopy allows for measurements of intermolecular interaction dynamics,^115^ so that exploratory biophysics measurements of nanopore-enabled glycan molecular recognition for identification and analysis can be used to assist molecular recognition engineering efforts. Second, nanoconfinement of molecules and complexes inside suitably sized nanopores (in contrast to presentation on open planar microarray surfaces) is a compelling parameter for tuning molecular-recognition-based sensing. While SiN_x_ nanopores are size-tunable,^85,104–107^ the size of the recognition agent has an impact on surface packing density and other factors. Lectins, antibodies, and even the Lambody class of GBPs are all large, multi-domain or multimeric proteins: the small size of the repurposed DNA binding protein GBPs^89^ may make them the preferred choice based solely on nanopore surface coating reasons. Of course, the small size would limit the potential glycan epitope/MICU it can potentially recognize (mono-, di- and trisaccharides), and so it may be necessary to develop methods to incorporate a mixture of different small GBPs on the surface of the same nanopore or in nanopore arrays for multifaceted glycan recognition.

## Conclusions

Nanopore glycomics has crossed the threshold of showing that a range of different glycans can be sensed by nanopores and, in curated sample sets, can be differentiated by length and by chemical composition. The keys to success have been combinations of well-characterized samples (underscoring the critical importance of developing glycan standards), high-contrast differences between less-well-characterized samples, nanopore surface-immobilized molecular recognition agent binding, and the use of machine learning approaches^59^ to maximize the information available from the resistive-pulse sensing experiments using sets of glycans. The tremendous scope and complexity of possible glycan structures and their widespread importance mean that exploratory and survey nanopore studies will remain important, as will nanopore studies targeting specific analysis tasks such as detecting impurities in glycan clinical therapeutics. However, advancing the field in a manner that can contend with the enormous complexity of glycan composition and analysis will require a systematic approach that tightly integrates nanopore design; glycan synthesis; molecular recognition use, study, and engineering; and experimental design and analysis—spanning atomistic simulations^116^ to careful control studies to machine learning^59^—that maximizes the information content when a nanopore is used to characterize a given glycan. As part of this effort, it is important to seek fundamental principles of molecular, device, and experimental designs while also seeking generality and robustness. Interdisciplinary efforts combining glycan synthesis and glycan recognition engineering with nanopore design and signal analysis will be essential. To spur such collaborative development, a diversity of approaches is vital, to deliver immediate results to a variety of different constituencies; provide iterative feedback and uncover key principles; and lay the foundation for meeting the aspirational goal of determining the total sequence of an unknown glycan at the single-molecule level. The tools and early results are in place to begin such interdisciplinary work in earnest to generate the exciting results that will catalyze further advances towards cracking the glycan code.
